# Factors associated with infertility in males and females in Qatar: A case-control study

**DOI:** 10.5339/qmj.2025.83

**Published:** 2025-09-23

**Authors:** Haya Alkuwari, Mohamed Ibrahim, Nour Darwish, Hamad Almaraghi, Alyaa AI-Wuhaili, Fatema Al-Muslamani, Mohamed Iheb Bougmiza, Tawanda Chivese, Mohamed Emara

**Affiliations:** 1 College of Medicine, Qatar University, Doha, Qatar; 2 Community Medicine Residency Program, Primary Health Care Corporation, Doha, Qatar; 3 Department of Science and Mathematics, School of Interdisciplinary Arts and Sciences, University of Washington Tacoma, Washington, United States of America *Email: ha2002868@qu.edu.qa

**Keywords:** Hypothyroidism, infertility, PCOS, Qatar, reproductive age group, risk factors, varicocele

## Abstract

**Background:**

Infertility is a rising global and regional concern, yet its associated factors remain underexplored in Qatar. This study investigated demographic, lifestyle, and medical factors linked to infertility, aiming to provide insights that can enhance reproductive healthcare strategies and patient outcomes in the region.

**Methods:**

A total of 998,328 eligible individuals from 27 Primary Health Care Centers in Qatar were identified for this case-control study. Data were collected between January 2016 and December 2022. Eligible individuals were aged 18 to 49 years with a documented fertility status. After 1:1 matching by age, gender, and nationality, the final sample included 51,542 participants (25,771 cases, 25,771 controls). Analyses were stratified by gender and nationality.

**Results:**

A total of 16,158 female cases and 9,613 male cases with infertility were identified and matched to fertile individuals. After multivariable logistic regression, in Qatari and non-Qatari females, polycystic ovarian syndrome was associated with higher odds of infertility (odds ratio [OR], 4.34 [95% CI, 3.36–5.61]; *p* < 0.001) and (OR, 5.54 [95% CI, 4.74–6.47]; *p* < 0.001), respectively. Uterine polyps showed a strong association in both groups (OR, 3.18 [95% CI, 2.21–4.59]; *p* < 0.001) and (OR, 6.05 [95% CI, 4.57–8.01]; *p* < 0.001), respectively. In both male groups, varicocele was associated with higher odds of infertility (OR, 39.46 [95% CI, 22.60–68.90]; *p* < 0.001) and (OR, 37.75 [95% CI, 26.32–54.16]; *p* < 0.001), respectively. Similarly, in erectile dysfunction (OR, 4.54 [95% CI, 3.32–6.21]; *p* < 0.001) and (OR, 6.59 [95% CI, 4.74–9.17]; *p* < 0.001), respectively.

**Conclusion:**

In females and males, reproductive diseases and other comorbidities were associated with higher odds of infertility in both Qatari and non-Qatari individuals. Future studies should investigate temporal associations and include additional risk factors to guide effective infertility prevention strategies.

## 1. INTRODUCTION

Infertility is a growing global health issue, affecting an estimated 186 million individuals worldwide, and contributing to significant personal, social, and economic burdens.^1^ According to the World Health Organization (WHO), infertility is defined as the failure to achieve a clinical pregnancy after 12 months or more of regular unprotected sexual intercourse.^1^ It can be further categorized into primary infertility, in which a person has never conceived, and secondary infertility, where conception has occurred in the past but not again despite trying for over a year.

The total fertility rate (TFR) has been decreasing worldwide since the 1950s.^1^ In the Middle East and North Africa region, the TFR has decreased from almost seven children per woman in 1950 to two and a half children per woman in 2019.^2^ Infertility prevalence was highest in Sub-Saharan Africa and Southern Asia, due to different reproductive conditions.^3^ However, this decrease in TFR is not solely due to infertility. Over the past 75 years, significant changes have occurred in family planning, contraceptive use, marriage trends, and societal roles. People are choosing to marry later in life, use advanced contraceptive methods, and there is greater access to family planning. Additionally, more women are pursuing higher education and actively participating in the workforce, leading to shifts in traditional family structures and reproductive choices.^4,5^

Males and females share several infertility risk factors, including non-modifiable factors such as age and congenital anomalies,^6^ and modifiable risk factors including body mass index (BMI) and smoking.^7^ Additionally, sexually transmitted infections, thyroid abnormalities, and diabetes impact fertility chances in both genders. However, some risk factors are unique to each gender.^8,9^ Male-specific factors include semen quality and reproductive system pathologies like varicoceles.^9,10^ Female-specific risk factors include irregular menstrual cycles, polycystic ovarian syndrome (PCOS), leiomyoma, and polyps.^7^

The impact of infertility includes a wide range of socio-cultural, emotional, physical, and financial problems.^11^ For couples with infertility, they may face increased marital conflicts and isolation. In some cultures, females with infertility may experience loss of their self esteeem, as fertility is often considered a key aspect of their identity and femininity, leading to feelings of shame and social isolation. Males with infertility may also experience loss of self esteem, as their ability to father children is commonly tied to notions of masculinity and strength, which may result in a diminished sense of self-worth and emotional distress.^11^ Despite this understanding, there is a lack of large-scale, population-based studies in Qatar that explore infertility risk factors across both genders and nationality groups.

This study aimed to identify and analyze demographic, lifestyle, and medical risk factors associated with infertility among males and females in Qatar, utilizing Primary Health Care Centers (PHCCs) databases to inform future reproductive health strategies. PHCCs in Qatar are governmental facilities and operate under the supervision of the Ministry of Public Health. Data from PHCCs was chosen, considering they are the first point of care for most residents in Qatar. The PHCCs serve over 1 million patients annually, and keep records of every visit, in addition to demographic data, diagnoses, and prescriptions.^12^

## 2. METHODS

### 2.1 Study Design

An analytical case-control study was performed to investigate the factors associated with infertility.

### 2.2 Study Settings, Duration, and Sample Size

Data of patients from all the 27 PHCCs in Qatar were used in this study. PHCCs were chosen as they serve as the first point of care for most residents and maintain detailed records of patient visits, demographics, diagnoses, and prescriptions. Participants were identified based on documented fertility status, and cases (with infertility) were matched with controls (without infertility) in a 1:1 ratio. Out of 998,328 eligible individuals, the final matched sample included 51,542 participants (25,771 cases and 25,771 controls). A formal a priori sample size calculation was not conducted. Instead, the final sample size (*n* = 51,542) was based on the total number of eligible cases of infertility identified from the PHCC dataset between January 2016 and December 2022 and successfully matched 1:1 with fertile controls. This comprehensive inclusion approach ensured maximum utilization of available data and enhanced statistical power. Given the large sample size, the study is sufficiently powered to detect modest effect sizes with high precision. Ethical approval was obtained from the PHCC Institutional Review Board (IRB; BUHOOTH-D-22-00011) and Qatar University IRB (2042542-1). Informed consent was waived due to the use of de-identified data.

### 2.3 Target Population

***2.3.1 Inclusion Criteria***

Cases: Any Qatari and non-Qatari male and female patients within the reproductive age (between 18–49 years old) who visited the PHCC between January 2016 to December 2022 and were diagnosed with infertility.

Controls: Any Qatari and non-Qatari male and female patients within the reproductive age (between 18–49 years old) who visited the PHCC between January 2016 to December 2022 for reasons other than infertility.

***2.3.2 Exclusion Criteria***

Cases: Male patients who underwent vasectomy or female patients who underwent hysterectomy. In addition, patients who were diagnosed with testicular or ovarian cancer and were receiving treatment for the diagnosis, such as chemotherapy and radiotherapy they may experience secondary reproductive failure and infertility.^13^

Controls: Patients with a diagnosis of infertility, patients who were previously managed for infertility, or those with a current pregnancy being a result of infertility management.

### 2.4 Exposures and Outcomes

Data collected included general demographic information, such as age, gender, nationality, average of the last three recorded heights in centimeters (cm), and weights in kilograms (kg) between the period of 2016 and 2022. According to WHO criteria, BMI was calculated as weight in kilograms divided by height in meters squared (kg/m^2^).^14^ We categorized BMI according to the Centers for Disease Control and Prevention criteria, with underweight defined as <18.5 kg/m^2^, normal as 18.5 to 24.9 kg/m^2^, overweight as 25.0 to 29.9 kg/m^2^, obese as 30.0 to 39.9 kg/m^2^, and severely obese as >40 kg/m^2^.^15^ System related factors were also collected including, cardiovascular system (cardiovascular diseases, hypertension, and dyslipidemia), reproductive system (history of irregular periods, history of polyps, PCOS, uterine fibroids/leiomyoma, endometriosis, pelvic inflammatory disease (PID), male genitourinary disorders (prostate hyperplasia), orchitis, history of varicocele, erectile dysfunction in males, respiratory system [asthma and chronic obstructive pulmonary disease (COPD)], endocrine system (diabetes mellitus, hyperthyroidism, and hypothyroidism), rheumatic diseases (arthritis and rheumatic heart disease), liver disorders and immunity disorders. Factors related to lifestyle patterns, such as smoking status and obesity, were requested. In addition, we received the median of the last three recorded hemoglobin and glycated hemoglobin (HbA1C) levels as laboratory records. The outcome measured in this study is infertility.

### 2.5 Statistical Analysis

The continuous variables were presented as means and standard deviations. The categorical variables were presented as and percentages. To analyze categorical variables, chi-squared tests were used for hypothesis testing. To analyze continuous data, independent t-tests were used to compare the means of two groups. We matched cases and controls in a 1:1 ratio for age, gender, and nationality to reduce confounding factors in the analysis. We then stratified by gender (male vs. female) and nationality (Qatari vs. non-Qatari) and adjusted for age. Four multivariable logistic regression models were used to assess the association between predictor factors and infertility. Variables that had the odds ratio (OR) close to 1 or did not add predictive value to the model in either nationality groups were removed. A separate model including all the factors assessed in the study is included as a Supplementary Table 1. The first model was used to assess the association of Qatari female infertility, and the second model was used for non-Qatari female infertility. The third model was used to assess the association of Qatari male infertility, and the fourth model was used for non-Qatari male infertility. ORs were reported, and 95% confidence intervals and exact *p*-values were reported. Exact p-values were reported. Analysis was performed using STATA SE 17.

## 3. RESULTS

### 3.1 Comparison of Characteristics of Cases and Controls

Table 1 shows the sociodemographic characteristics of cases and controls. Since age, gender, and nationality were matched, the cases and controls were equally distributed in these variables. The mean age was 35.7 (SD, 6.2) years in females and 37.4 (SD, 6.0) years in males. Regarding nationality, 31.1% of females and males were Qatari. Females with infertility had a higher percentage of smokers (1.6%) compared to (1.3%) of those without infertility, with a statistically significant p-value (*p* = 0.009). Similarly, males with infertility had a higher percentage of smokers (22.6%) compared to 17.5% of males without infertility, which was statistically significant (*p* < 0.001).

### 3.2 Factors Associated With Infertility in Females

Table 2 shows the results of the multivariable logistic regression for factors associated with infertility in Qatari and non-Qatari females. All the reproductive factors showed strong associations with infertility in both Qatari and non-Qatari females. Excluding PID, all the risk factors showed stronger associations in non-Qatari females compared to Qatari females. In Qatari females, PCOS showed the strongest association with a 4-fold increase in the odds of infertility (OR, 4.34 [95% CI, 3.36–5.61]; *p* < 0.001). In non-Qatari females, the strongest association was uterine polyps (OR, 6.05 [95% CI, 4.57–8.01]; *p* < 0.001).

Regarding the non-reproductive system-related diseases, only a few retained strong associations with infertility in the adjusted analysis. Diabetes showed a strong association in both Qatari and non-Qatari females (OR, 1.32 [95% CI, 1.16–1.50]; *p* < 0.001) and (OR, 1.69 [95% CI, 1.50–1.90]; *p* < 0.001), respectively. Similarly, hypothyroidism also revealed a strong association in both Qatari and non-Qatari females (OR, 1.78 [95% CI, 1.60–1.97]; *p* < 0.001) and (OR, 2.07 [95% CI, 1.90–2.24]; *p* < 0.001), respectively. Smoking and liver disorders showed a strong association with infertility only in non-Qatari females (OR, 1.56 [95% CI, 1.24–1.95]; *p* < 0.001) and (OR, 1.60 [95% CI, 1.02–2.52]; *p* < 0.041), respectively. On the other hand, immunity disorders were the only non-reproductive risk factor that showed a strong association in Qatari females (OR, 3.66 [95% CI, 1.17–11.41]; *p* = 0.025).

### 3.3 Factors Associated With Infertility in Males

Table 3 shows the results of the multivariable logistic regression for factors associated with infertility in Qatari and non-Qatari males. Regarding the reproductive factors, varicocele, erectile dysfunction, and male genitourinary disorders showed strong associations with infertility in both Qatari and non-Qatari males. Varicocele showed the strongest association in both groups (OR, 39.46 [95% CI, 22.60–68.90]; *p* < 0.001) and (OR, 37.75 [95% CI, 26.32–54.16]; *p* < 0.001), respectively. However, orchitis showed a strong association with infertility in only Qatari males (OR, 9.36 [95% CI, 1.16–75.49]; *p* < 0.036).

Regarding the non-reproductive system-related diseases, diabetes, hypothyroidism, obesity, and asthma or COPD retained strong associations with infertility in both Qatari and non-Qatari males in the adjusted analysis. Smoking (OR, 1.40 [95% CI, 1.27–1.56]; *p* < 0.001), dyslipidemia (OR, 1.56 [95% CI, 1.37–1.78]; p < 0.001), and liver disorders (OR, 2.95 [95% CI, 1.85–4.71]; *p* < 0.001), showed strong association with infertility only in non-Qatari males.

## 4. DISCUSSION

In this study of 51,542 participants at PHCC in Qatar from January 2016 to December 2022, we identified several infertility risk factors. Reproductive disorders, hypothyroidism, and diabetes were linked to higher odds of infertility in both genders across Qatari and non-Qatari individuals. Obesity and asthma/COPD increased infertility risk in both Qatari and non-Qatari males, while orchitis was limited to Qatari males. Immunity disorders were associated with increased risk in Qatari females, liver disorders, and dyslipidemia were associated with infertility in non-Qatari females.

Our study revealed a heightened risk of infertility in both Qatari and non-Qatari females due to reproductive factors. PCOS contributed the most to our model in Qataris and second most in non-Qataris (4-fold higher odds) and (6-fold higher odds), respectively. In agreement with our results, a study in China revealed that females with PCOS have around 7 times the increase in the odds of infertility compared to those without PCOS (OR, 6.72 [95% CI, 1.79–7.39]; *p* value ≤ 0.001).^16^ The pathogenesis of PCOS involves the failure to produce a mature egg due to hormonal abnormalities such as high androgens.^17^ Furthermore, our study found that endometrial polyps pose a heightened risk of infertility in Qatari and non-Qatari females 4-fold higher odds and 6-fold higher odds, respectively. In alignment with our findings, a study conducted in Saudi Arabia found that 9% of women with infertility had endometrial polyps.^18^ The exact mechanism of infertility in endometrial polyps is unknown, but it is believed to be due to mechanical obstruction affecting sperm movement and failure of implantation.^19^ Our study confirmed that endometriosis was associated with a higher risk of infertility. A study in the United States mentioned that around 50% of females with endometriosis are infertile.^20^ Endometriosis is thought to disrupt oocyte release, gamete transport, and implantation through the presence of inflammatory cells.^21^ Moreover, our study validated that irregular menstruations increase the risk of infertility, as previously established.^22–24^ Additionally, our study confirmed that leiomyoma increases the risk of infertility, as previously investigated.^16,25^ Several theories suggest that leiomyoma disrupts the local anatomy of myometrium and endometrium, causing impaired gamete transport and embryo implantation.^26^

Our study confirmed a link between diabetes and female infertility in both Qataris and non-Qataris, similar to findings from other studies in Qatar.^5^ The ovaries contain insulin receptors. Persistent exposure to high insulin levels can potentially result in increased production of ovarian androgens, leading to infertility.^27^ Our study found that hypothyroidism was strongly associated with female infertility in Qataris and non-Qataris. Our findings were supported by another hospital-based case control study, which found that hypothyroidism was reported significantly higher among women with infertility (19.1%), compared to controls (11.0%).^22^ Hypothyroidism can cause anovulatory cycles, luteal phase defects, high prolactin levels, and sex hormone imbalances.^5^ Immunity disorders were associated with a 3.66 increase in odds of infertility in Qatari females with a statistically significant p-value of 0.025; however, further research is needed to support this association.

Our study confirmed that varicocele was associated with a higher risk of infertility in both Qataris and non-Qatari men 40-fold higher odds and 38-fold higher odds, respectively. Varicocele is the most correctable cause of male infertility.^28^ The pathogenesis of varicocele induced infertility remains controversial, however oxidative stress is thought to play a key role.^28^ Correlating with our study, a meta-analysis consisting of 12 studies published from 2006 to 2021 assessing the effect of varicoceles on sperm DNA fragmentation showed a statistically significant difference in DNA fragmentation index between varicocele group and healthy controls with a standardized mean difference of 1.4 (SMD, 1.40 [95% CI, 0.83–1.98]; *p* < 0.0001) which suggests that varicoceles can lead to sperm DNA damage therefore affecting male fertility.^29^ In our study, orchitis was found to increase the risk of infertility by 9.36-fold in Qatari males only. According to a study in China, 30% of mumps orchitis in post-pubertal males suffer from infertility or subfertility. The study stated that mumps orchitis increases the risk of infertility through the arrest of spermatogenesis.^30^ According to a study conducted in Brazil, autoimmune orchitis increases the risk of infertility through the presence of anti-sperm antibodies.^31^ Further research is needed to investigate the association between orchitis and infertility in Qatari males. Consistent with prior research, our findings indicate a link between erectile dysfunction and infertility in Qataris and non-Qataris 4.5-fold higher odds and 6.6-fold higher odds, respectively. Erectile dysfunction and premature ejaculation are considered indirect causes of infertility, as sexual dysfunction results in limited regular sexual activity. A recent meta-analysis of eight studies found a remarkably higher prevalence of erectile dysfunction in men with infertility compared to controls (OR, 2.66 [95% CI, 1.69−4.19]; *p* < 0.001).^32^ In agreement with the literature, male genitourinary disorders increase the risk of infertility in Qataris and non-Qataris. For instance, autoimmune prostatitis is associated with infertility through Th1 cells and other leukocytes affecting sperm and semen quality.^33^ Another study found that chronic epididymitis is associated with low sperm motility.^34^

In accordance with other studies, our study demonstrated that obesity and diabetes had a strong association with male infertility in Qataris and non-Qataris (Table 3). The link between obesity and male infertility is not well understood, but it could be due to endocrine disturbances that could impair spermatogenesis.^5,35^ Regarding diabetes, it is hypothesized that impairment of glucose metabolism affects both sperm motility and maturation.^5^ In our study, dyslipidemia was found to increase the odds of infertility by 1.56 times in non-Qataris; however, the effect of abnormal levels of lipids has not been thoroughly explored in other literature.^34^ The lack of link between dyslipidemia and infertility in Qataris may be due to better access to dyslipidemia control compared to non-Qataris.^36^ In line with prior research, hypothyroidism has been linked to infertility in both Qatari and non-Qatari males. This connection could be attributed to a decrease in sperm vitality due to increased oxidative stress.^37^ Our study also showed the association between asthma/COPD and infertility in Qatari and non-Qatari males 1.2-fold higher odds and 1.4-fold higher odds, respectively, In a prospective study of 90 men with moderate-to-severe COPD, 74% experienced at least one sexual dysfunction—most commonly erectile dysfunction (72%)—with low testosterone independently associated with erectile dysfunction.^38^

### 4.1 Strengths

In terms of the study’s strengths, this research is unique in measuring infertility risk factors in both sexes in Qataris and non-Qataris. This allows for the development of preventative strategies. The study’s large sample size of 51,542 participants provides high statistical power.

### 4.2 Weaknesses

A major weakness of this study is the broad classification of participants as Qatari and non-Qatari, which overlooks the diverse ethnic and cultural backgrounds within the non-Qatari group. Given the large sample size, a more detailed stratification by nationality could reveal important insights related to ethnicity and cultural factors influencing infertility. The current approach may dilute meaningful associations and limit the study’s interpretability. Additionally, the lack of a temporal relationship between dependent and independent variables makes it difficult to infer causality, as Hill’s criteria require further longitudinal data. The absence of key infertility indicators, such as sexually transmitted diseases, sperm analysis, and drug use, also limits the predictive strength of the model.

## 5. CONCLUSION

In conclusion, the current study identified associations between hypothyroidism, male genitourinary disorders, erectile dysfunction, varicocele, PCOS, and other risk factors with established pathogenesis leading to infertility. Furthermore, our study identified certain risk factors associated with infertility, particularly immune disorders in females and orchitis in males. This is one of the largest population-based studies on infertility conducted in the Middle East, using comprehensive real-world data from Qatar’s national primary health care system. The study provides novel insights into risk factors across both Qatari and non-Qatari populations, stratified by sex, and highlights differences in risk patterns that may guide targeted interventions. Further studies should focus on investigating temporal relationships and including additional risk factors to address the rising cases of infertility and develop preventative strategies in the future.

## LIST OF ABBREVIATIONS

**Table:** 

BMI	Body mass index
CMED	College of Medicine at Qatar University
COPD	Chronic obstructive pulmonary disease
DPM	Department of Population Medicine
HbA1c	Glycated hemoglobin
OR	Odds ratio
PCOS	Polycystic ovarian syndrome
PHCC	Primary Health Care Center
PID	Pelvic inflammatory disease
SD	Standard deviation
TFR	Total fertility rate
WHO	World Health Organization

## ACKNOWLEDGMENTS

The authors gratefully acknowledge the support of the Primary Health Care Corporation (PHCC), the Department of Population Medicine (DPM), and the College of Medicine at Qatar University (CMED) for their valuable contributions to this research.

## CONFLICT OF INTEREST

All authors declare no conflict of interest.

## Figures and Tables

**Table 1.** Demographic and lifestyle characteristics of participants by fertility status.

		Female			Male		
Factor	Level	without infertility	with infertility	*p* value	without infertility	with infertility	*p* value
*N*		16,158	16,158	-	9,613	9,613	-
Age, mean (SD)		35.7 (6.2)	35.7 (6.2)	1.00	37.4 (6.0)	37.4 (6.0)	1.00
Age groups	18–29 years	2723 (16.9)	2723 (16.9)	1.00	897 (9.3)	897 (9.3)	1.00
	30–39 years	8895 (55.1)	8895 (55.1)		5225 (54.4)	5225 (54.4)	
	40–49 years	4540 (28.1)	4540 (28.1)		3491 (36.3)	3491 (36.3)	
Nationality	Non-Qatari	11134 (68.9)	11134 (68.9)	1.00	6622 (68.9)	6622 (68.9)	1.00
	Qatari	5024 (31.1)	5024 (31.1)		2991 (31.1)	2991 (31.1)	
Smoking	No	15952 (98.7)	15896 (98.4)	0.009	7931 (82.5)	7438 (77.4)	<0.001
	Yes	206 (1.3)	262 (1.6)		1682 (17.5)	2175 (22.6)	
Body mass index	Normal weight	2533 (23.8)	2869 (19.9)	<0.001	927 (21.3)	1323 (19.3)	0.003
	Underweight	164 (1.5)	135 (0.9)		64 (1.5)	68 (1.0)	
	Overweight	3670 (34.5)	4833 (33.6)		1716 (39.3)	2714 (39.7)	
	Obese	3721 (34.9)	5708 (39.7)		1456 (33.4)	2353 (34.4)	
	Severely obese	565 (5.3)	846 (5.9)		198 (4.5)	382 (5.6)	

Matched for age, gender, and nationality, hence why p-value = 1.00. Underweight <18.5 kg/m^2^, normal weight 18.5 to 24.9 kg/m^2^, overweight 25.0 to 29.9 kg/m^2^, obese 30.0 to 39.9 kg/m^2^, and severely obese >40 kg/m^2^.

**Table 2.** Factors associated with infertility in females—multivariable logistic regression.

	Qatari female (*N* = 5,024)			Non-Qatari female (*N* = 11,134)		
	Odds ratio	*p* value	95% CI	Odds ratio	*p* value	95% CI
PCOS	4.34	<0.001	3.36–5.61	5.54	<0.001	4.74–6.47
Immunity disorders	3.66	0.025	1.17–11.41	1.09	0.869	0.38–3.10
Uterine polyps	3.18	<0.001	2.21–4.59	6.05	<0.001	4.57–8.01
PID	2.98	<0.001	1.81–4.91	1.94	<0.001	1.43–2.63
Endometriosis	2.63	<0.001	1.70–4.07	5.10	<0.001	3.82–6.82
Irregular menstrual cycle	2.18	<0.001	1.99–2.38	2.87	<0.001	2.70–3.06
Hypothyroidism	1.78	<0.001	1.60–1.97	2.07	<0.001	1.90–2.24
Leiomyoma	1.34	0.036	1.02–1.77	2.22	<0.001	1.87–2.64
Diabetes	1.32	<0.001	1.16–1.50	1.69	<0.001	1.50–1.90
Liver disorders	1.28	0.472	0.66–2.49	1.60	0.041	1.02–2.52
Smoking	0.72	0.144	0.47–1.12	1.56	<0.001	1.24–1.95
Constant	0.63	<0.001	0.57–0.69	0.48	<0.001	0.44–0.52

In each model (Qatari females and non-Qatari females), a multivariable logistic regression was carried out with the outcome as infertility (yes/no), and all the exposures in the table were adjusted for age. PCOS stands for polycystic ovarian syndrome, and PID stands for pelvic inflammatory disease. Immunity disorders include human immunodeficiency virus (HIV) disease, immunodeficiency with predominantly antibody defects, immunodeficiency associated with other major defects, common variable immunodeficiency, sarcoidosis, and other disorders involving an immune mechanism. Liver disorders include chronic viral hepatitis, chronic hepatitis, and hepatic failure. The “Constant” refers to the model intercept, representing the odds of infertility when all predictors are at their reference values (coded as 0).

**Table 3.** Factors associated with infertility in males—multivariable logistic regression.

	Qatari male (*N* = 2,991)			Non-Qatari male		
	Odds ratio	*p* value	95% CI	Odds ratio	*p* value	95% CI
Varicocele	39.46	<0.001	22.60–68.90	37.75	<0.001	26.32–54.16
Orchitis	9.36	0.036	1.16–75.49	0.71	0.429	0.31–1.64
Erectile dysfunction	4.54	<0.001	3.32–6.21	6.59	<0.001	4.74–9.17
Male GU disorders	3.17	<0.001	2.43–4.12	6.50	<0.001	5.16–8.18
Hypothyroidism	1.79	<0.001	1.32–2.42	2.20	<0.001	1.71–2.83
Obesity	1.55	<0.001	1.33–1.80	1.73	<0.001	1.49–2.02
Diabetes	1.42	<0.001	1.18–1.71	1.49	<0.001	1.29–1.71
Asthma or chronic obstructive pulmonary disease	1.20	0.048	1.00–1.44	1.44	<0.001	1.20–1.72
Dyslipidemia	1.06	0.558	0.87–1.29	1.56	<0.001	1.37–1.78
Smoking	0.90	0.112	0.80–1.02	1.40	<0.001	1.27–1.56
Liver disorders	0.85	0.704	0.36–2.00	2.95	<0.001	1.85–4.71
Constant	0.56	<0.001	0.48–0.64	0.56	<0.001	0.48–0.66

In each model (Qatari males and non-Qatari males), a multivariable logistic regression was carried out with the outcome as infertility (yes/no), and all the exposures in the table were adjusted for age. Male GU Disorders stands for male genitourinary disorders, which indicate benign prostatic hyperplasia, inflammatory conditions of male genital organs, and other specified male genital disorders. Liver disorders indicate chronic viral hepatitis, chronic hepatitis, and hepatic failure. The “Constant” refers to the model intercept, representing the odds of infertility when all predictors are at their reference values (coded as 0).

## Supplementary Material

**Supplementary Table 1.** Risk factors in females and males by fertility status.

	Female			Male		
Factor	Fertile	Infertile	*p* value	Fertile	Infertile	*p* value
*N*	16158	16158	-	9613	9613	-
Smoking	206 (1.3)	262 (1.6)	0.009	1682 (17.5)	2175 (22.6)	< 0.001
Hb	11.9 (1.3)	12.1 (1.2)	<0.001	15.0 (1.2)	14.8 (1.2)	<0.001
Glycated hemoglobin (HbA1c), mean (SD)	5.5 (0.8)	5.5 (0.8)	<0.001	5.9 (1.3)	5.8 (1.2)	0.20
Diabetes	1052 (6.5)	1946 (12.0)	<0.001	687 (7.1)	1226 (12.8)	<0.001
Hypertension	821 (5.1)	1145 (7.1)	<0.001	724 (7.5)	1143 (11.9)	<0.001
Cardiovascular diseases	58 (0.4)	82 (0.5)	0.042	108 (1.1)	202 (2.1)	<0.001
Dyslipidemia	936 (5.8)	1446 (8.9)	<0.001	767 (8.0)	1341 (13.9)	<0.001
Asthma or COPD	1037 (6.4)	1496 (9.3)	<0.001	512 (5.3)	777 (8.1)	<0.001
Rheumatic diseases	71 (0.4)	108 (0.7)	0.006	17 (0.2)	41 (0.4)	0.002
Immunity disorders	10 (0.1)	25 (0.2)	0.011	10 (0.1)	25 (0.3)	0.011
Liver disorders	49 (0.3)	86 (0.5)	0.001	39 (0.4)	103 (1.1)	<0.001
Obesity	2327 (14.4)	3571 (22.1)	<0.001	683 (7.1)	1314 (13.7)	<0.001
Hyperthyroidism	286 (1.8)	426 (2.6)	<0.001	61 (0.6)	86 (0.9)	0.038
Hypothyroidism	1870 (11.6)	3774 (23.4)	<0.001	171 (1.8)	405 (4.2)	<0.001
**Female reproductive system disorders**						
Irregular menstrual cycles	3444 (21.3)	7657 (47.4)	<0.001	-	-	-
Uterine polyps	99 (0.6)	622 (3.8)	<0.001	-	-	-
Polycystic ovarian syndrome	277 (1.7)	1807 (11.2)	<0.001	-	-	-
Leiomyoma	295 (1.8)	820 (5.1)	<0.001	-	-	-
Endometriosis	87 (0.5)	459 (2.8)	<0.001	-	-	-
Pelvic inflammatory disease	89 (0.6)	267 (1.7)	<0.001	-	-	-
**Male reproductive system disorders**						
Varicocele	-	-	-	44 (0.5)	1667 (17.3)	<0.001
Erectile dysfunction	-	-	-	95 (1.0)	646 (6.7)	<0.001
Orchitis	-	-	-	12 (0.1)	55 (0.6)	<0.001
Male genitourinary disorders	-	-	-	173 (1.8)	1284 (13.4)	<0.001

Male genitourinary disorders indicate benign prostatic hyperplasia, inflammatory conditions of male genital organs, other specified male genital disorders. *P* values are statistically significant due to the large sample size.
